# A Fast Humidity Sensor Based on Li^+^-Doped SnO_2_ One-Dimensional Porous Nanofibers

**DOI:** 10.3390/ma10050535

**Published:** 2017-05-16

**Authors:** Min Yin, Fang Yang, Zhaojie Wang, Miao Zhu, Ming Liu, Xiuru Xu, Zhenyu Li

**Affiliations:** 1 School of Advanced Materials, Peking University Shenzhen Graduate School, Shenzhen 518055, China; zhumiao@pkusz.edu.cn (M.Z.); liuming@pkusz.edu.cn (M.L.); 2 Institute of Chemical Materials, China Academy of Engineering Physics, Mianyang 621900, China; yf6897@163.com; 3 Institute for Frontier Materials, Deakin University, Geelong, Victoria 3216, Australia; zhenyuli@deakin.edu.au; 4 Changchun Institute of Applied Chemistry, Chinese Academy of Sciences, 5625 Renmin Street, Changchun 130022, China; ymin1988@126.com; 5 Alan G. MacDiarmid Institute, Jilin University, Changchun 130012, China; 6 College of Science, China University of Petroleum, Qingdao 266580, China; wangzhaojie@upc.edu.cn

**Keywords:** humidity sensor, electrospun porous nanofibers, lithium doping, response-recovery behavior

## Abstract

One-dimensional SnO_2_- and Li^+^-doped SnO_2_ porous nanofibers were easily fabricated via electrospinning and a subsequent calcination procedure for ultrafast humidity sensing. Different Li dopant concentrations were introduced to investigate the dopant’s role in sensing performance. The response properties were studied under different relative humidity levels by both statistic and dynamic tests. The best response was obtained with respect to the optimal doping of Li^+^ into SnO_2_ porous nanofibers with a maximum 15 times higher response than that of pristine SnO_2_ porous nanofibers, at a relative humidity level of 85%. Most importantly, the ultrafast response and recovery time within 1 s was also obtained with the 1.0 wt % doping of Li^+^ into SnO_2_ porous nanofibers at 5 V and at room temperature, benefiting from the co-contributions of Li-doping and the one-dimensional porous structure. This work provides an effective method of developing ultrafast sensors for practical applications—especially fast breathing sensors.

## 1. Introduction

Humidity sensors are of great importance in many fields, including environmental monitoring, industrial production, agricultural planting, aviation, and medical and chemical monitoring [1,2,3]. Until now, various transduction techniques have been used to develop humidity sensors, such as capacitance [4], impedance [5], optical fiber [6,7], and surface acoustic wave (SAW) [8]. Nanostructured metal oxides are ideal materials for the fabrication of humidity sensors because of the ability to tailor their surface and charge-transport properties, as well as their chemical and physical stability and high mechanical strength [9,10,11]. Remarkably, as one of the most important typical n-type metal oxide semiconductors, SnO_2_ materials have attracted much attention as chemical sensing materials but have suffered from the obvious drawbacks of low conductivity, long response/recovery time, and narrow measuring range [8,12,13]. Especially, the slow response and recovery speed hindered the applications of SnO_2_-structured materials as humidity sensors.

The response of SnO_2_-based humidity sensors depends upon reactions between water molecules and SnO_2_ surfaces, which have stimulated the interest of researchers in tailoring the microstructure and morphology of SnO_2_ nanostructures [14,15]. One-dimensional (1D) nanostructures, such as nanowires, nanorods, and nanobelts, have attracted much attention, since, benefiting from their geometric advantages, they can make aimed molecule access easy and provide excellent electron transport and efficient response and recovery properties, [13,16,17,18]. Wang et al. synthesized high-yield SnO_2_ nanowires that demonstrate very high response performances on humidity sensing and a short reaction speed [16]. One-dimensional SnO_2_/TiO_2_ heterostructures were also successfully synthesized through the hydrothermal assembly of single-crystalline SnO_2_ nanocubes on TiO_2_ electrospun nanofibers, where the response and recovery times could reach ~2.4 s and ~30.2 s, respectively [19]. The SnO_2_ nanowire networks made from 1-D nanostructures were synthesized by a simple and versatile flame transport synthesis approach, exhibiting promising sensing performances [14]. Additionally, another approach for enhancing the sensing properties of SnO_2_ lies in its modification with metal nanoparticles, such as Ag, Fe, and Cu [20,21,22]. M. Sabarilakshmi synthesized W-doping SnO_2_ nanopowders and found that W-doping improves sensitivity along with a better response (38 s) and recovery time (25 s) toward open air at room temperature, leading to the synergic electronic interactions between the dispersed W and the characteristic formation of the SnO_2_ nanoparticles [23]. Ag-SnO_2_/SBA-15 also exhibited ultrahigh sensitivity over the entire RH range, along with rapid response (5 s) and recovery times (8 s), relatively low hysteresis (0.9%), and excellent stability (1.1%) in the 11%–98% RH range [24]. However, up to now, the response and recovery times of the reported sensors, even based on the nanostructured materials, are usually beyond 5 s (the time should be no more than 5 s in fast breathing sensors), which hinders ultrafast sensing applications [15]. 

In our work, in order to achieve the ultrafast humidity sensors, we provided a combined strategy of structured construction and doping. The main focus in our work was directed toward the design of a novel additive and structure of 1D nanostructured SnO_2_ that can increase and activate the interaction between sensing materials and moisture surroundings. Li^+^-doped SnO_2_ porous nanofibers (PNFs) were developed as a high-efficiency and ultrafast humidity sensor via electrospinning for the very first time. Excellent humidity sensing properties such as high sensitivity, especially fast response-recovery behavior (within 1 s), were achieved with the Li^+^-doped SnO_2_ 1D porous nanofibers. We thus offer here an effective approach toward an understanding, and the design, of SnO_2_-based humidity sensing materials.

## 2. Results and Discussion

### 2.1. Morphological and Structural Characteristics

**Figure 1:**
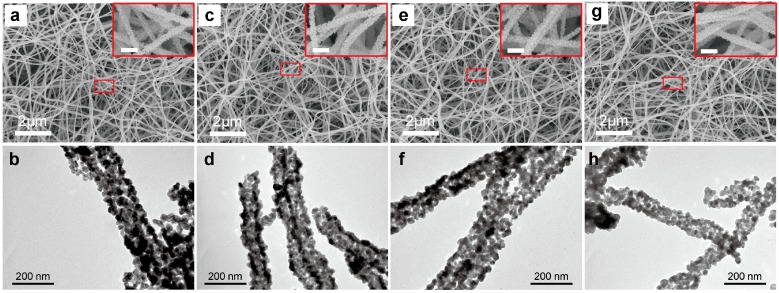
Scanning electron microscope images (**a**,**c**,**e**,**g**) and transmission electron microscope images (**b**,**d**,**f**,**h**) images of (**a**,**b**) pristine SnO_2_ porous nanofibers as well as (**c**,**d**) 1.0 wt %, (**e**,**f**) 4.0 wt %, (**g**,**h**) 8.0 wt % Li^+^-doped SnO_2_ porous nanofibers prepared by electrospinning and post-calcination at 600 °C in air for 5 h. The insets are high-resolution SEM images (Scale bar: 200 nm). The pristine SnO_2_ porous nanofibers and Li^+^-doped SnO_2_ porous nanofibers showed a porous structure. The Li^+^-doped SnO_2_ porous nanofibers can be controlled by changing the ratio of lithium chloride hydrate in precursors.

A scanning electron microscope and transmission electron microscope were used to characterize the morphologies of pristine SnO_2_ and Li^+^-doped SnO_2_ PNFs with different amounts of Li^+^ by electrospinning and post-calcination at 600 °C, as shown in Figure 1. All the as-prepared pristine SnO_2_ and the doped SnO_2_ display a uniform fiber-like structure. Simultaneously, the PVP will be removed with the post-calcination procedure, which results in the formation of high porosity and bended fibrous morphologies. The average diameters of the final nanofibers range from 80 to 100 nm, and the small nanocrystals composing these nanofibers are 10–20 nm. The large voids between the adjacent fibers and small pores within the final fibers will provide an easy pathway for water molecules to penetrate easily into the whole membranes, benefiting the humidity sensing properties. It can be concluded that SnO_2_ with a porous fiber-like morphology was successfully synthesized in this study via a simple electrospinning technique.

**Figure 2:**
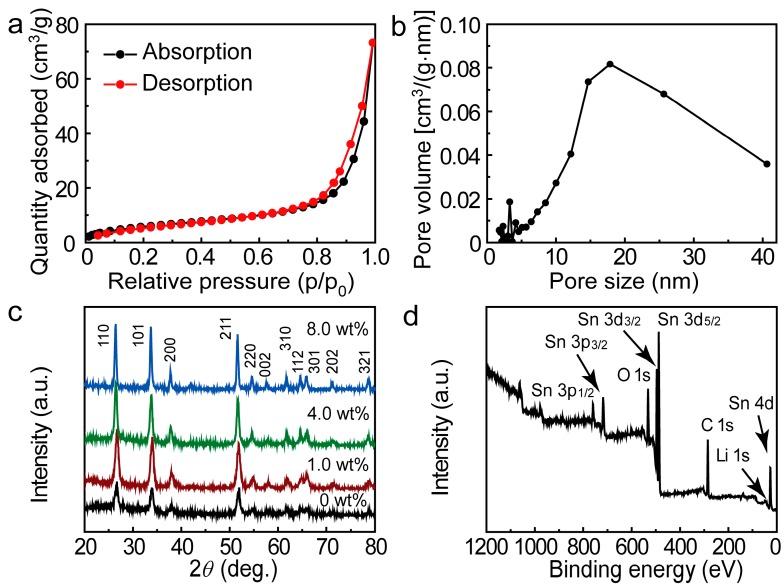
(**a**) Nitrogen adsorption/desorption isotherms; (**b**) DFT pore-size distribution plot of 1.0 wt % Li^+^-doped SnO_2_ porous nanofibers; (**c**) XRD patterns of pristine and Li^+^-doped SnO_2_ porous nanofibers; (**d**) XPS full spectrum of 1.0 wt % Li^+^-doped SnO_2_ porous nanofibers, which confirmed the presence of Li 1s (~55.0 eV) in the doping sample.

The porosity of the as-prepared porous nanofibers was then studied by nitrogen adsorption and desorption isotherms (ASAP 2020 HD88, Micromertitics instruments, Norcross, GA, USA). Based on the Barret-Joyner-Halenda (BJH) method and the adsorption branch of the nitrogen isotherm, the calculated pore size distribution indicates two correspondingly pore size distribution centers at about 3.2 nm and 17.9 nm. The BET surface area of the as-prepared nanofibers is calculated to be 22.53 m^2^/g (shown as Figure 2a,b). Figure 2c shows the XRD patterns of the as-prepared SnO_2_ PNFs with different Li doping levels. All of them display clear reflections from (110), (101), (200), (111), (210), (211), and (220) crystallographic planes of the rutile structure SnO_2_ crystalline phase (JCPDS 41-1445) [14,25]. No phase ascribed to Li oxides can be seen, indicating the possible entrance of Li into tin oxide lattice, which can be deduced from the lattice change of SnO_2_. As the Li dopant increases, the diffraction peaks of SnO_2_ were found to shift left, indicating the increased lattice constant by Li doping. To confirm the existence of Li in the Li^+^-doped SnO_2_ PNFs, an XPS spectrum was used to investigate the composition of the doping sample (shown in Figure 2d), which confirm the presence of Li 1s (~55.0 eV) [26] in the doping sample, as well as the presence of Sn 4d, Sn 3d_3/2_, Sn 3d_5/2_, Sn 3p_1/2_, Sn 3p_3/2_, C 1s, and O 1s [27].

### 2.2. Humidity Sensing Properties

**Figure 3:**
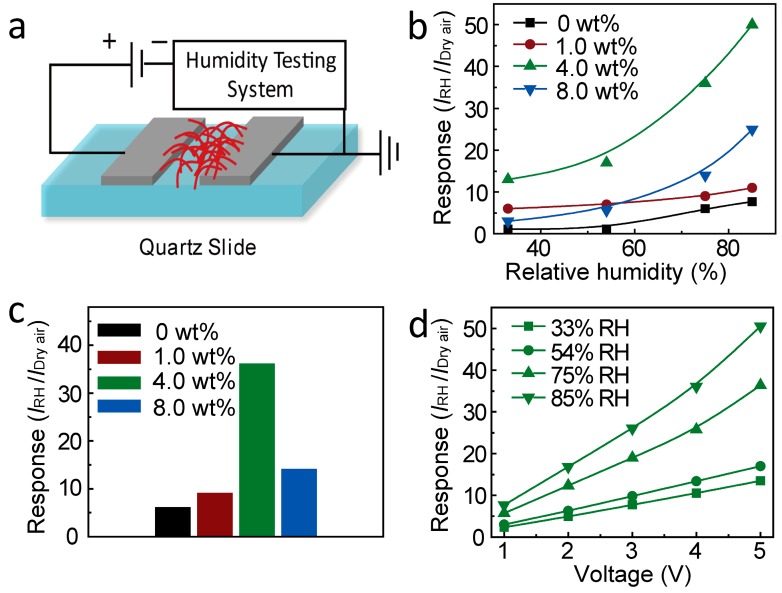
(**a**) Schematic illustration of the SnO_2_ porous nanofiber-based sensor; (**b**) the response vs. the relative humidity (RH) of different doping levels of Li^+^-doped SnO_2_ porous nanofibers at 5 V under room temperature; (**c**) the response comparison of different Li^+^-doped SnO_2_ porous nanofibers under 75% relative humidity; (**d**) the response sensitivity–driving voltage properties for 4.0 wt % Li^+^-doped SnO_2_ porous nanofiber sensors under various relative humidity levels.

**Figure 4:**
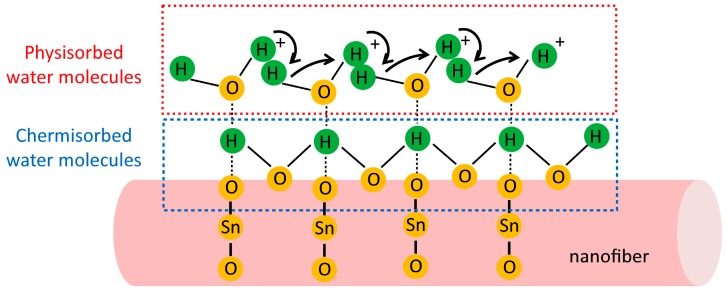
Schematic representation or the benefit of the one-dimensional characteristic structure of SnO_2_ porous nanofibers on humidity sensing performance.

The SnO_2_-based humidity sensor was then constructed by depositing a pair of Al electrodes, as illustrated in Figure 3a. The response of SnO_2_ PNF-based sensors at different humidity levels is defined as the ratio of the measured currents at selected RH value and that under dry air. In other words, it can be estimated as S = *I*_RH_/*I*_dry air_ [28], where the *I*_RH_ is the current of the sensor at the selected humidity level, and *I*_dry air_ is the current of the sensor at dry air condition. Figure 3b shows the response properties of different doping levels of Li^+^-doped SnO_2_ PNFs under different RH values. For the pristine SnO_2_ PNFs, the responses are 1.1, 1.2, 7.2, and 10.0 at the 33%, 54%, 75%, and 85% RH, respectively. The relatively low response, especially under low RH, e.g., 33% and 54%, is limited mainly by a poor adsorption ability of water molecules. Obviously, the water concentration in air has a strong influence on the humidity response of SnO_2_. The basic humidity sensing mechanism on the SnO_2_ is explained by the sorption of water molecules onto the surface, which is usually described by the condensation of water molecules onto the sensor surface, inducing the proton conduction and consequently causing a change in the net conductivity of the sensor [29]. At a relatively low RH value, the chemisorption occurs to form two surface hydroxyls per water molecule [30]. With increasing humidity levels, the physisorption of water molecules takes place, resulting in capillary condensation and conduction by the Grotthuss transport mechanism [31], further enhancing the humidity sensing properties. Therefore, humidity sensing was detected as increasing with the increasing RH value. The schematic representation of SnO_2_ PNFs on the humidity sensing performance is illustrated in Figure 4.

As Li^+^ was introduced to SnO_2_, the response sensitivity was obviously increased, which can be inferred from the curves in Figure 3b. A response comparison of different Li^+^-doped SnO_2_ PNFs under 75% RH value can be made based on Figure 3c, which clearly indicates the volcano-shape of the response with the Li doping content. The humidity response first increased as the Li^+^ doping content increased from 1.0 to 4.0 wt %, and then decreased as the Li^+^ doping content continued increasing to 8.0 wt %. The 4.0 wt % doped SnO_2_ showed the best response sensitivity, with values of 13.4, 17.0, 36.3, and 50.5 at 33%, 54%, 75%, and 85% RH, respectively, which is 5 times higher than that of the pristine SnO_2_ PNFs at high RH values of 75% and 85%, and around 15 times higher than that of the pristine PNFs at low RH values of 33% and 54%. 

The enhanced response can be mainly attributed to the alkaline Li^+^ bonding interface to create a strong adhesion to water molecules, which can effectively induce a current change [32]. The similar enhanced sensing performance was also reported for Li^+^-TiO_2_ nanofibers, which were demonstrated to act as a highly sensitive humidity sensor [17]. In our Li-SnO_2_ system, the responses of doped sensing nanofibers could be enhanced, even at low RH levels, also proving the important tuning ability of water molecular adsorption of Li doping on SnO_2_. However, the Li doping has optimal content. As the doping level increased to 8.0 wt %, worse performance was unfortunately detected, which may be attributed to the excess of the conduction carriers (Li^+^ ions) resulting in an increased value of *I*_dry air_ and the consequently a declined response value (*I*_RH_/*I*_dry air_). However, the humidity response is still higher than that of the pristine SnO_2_ PNFs. Figure 3d shows the response-driving voltage properties of the 4.0 wt % Li^+^-doped SnO_2_ PNFs sensor under various relative humidity levels. The linear behavior of the humidity response with driving voltage suggests optimum ohmic contact between SnO_2_ and the Al electrodes. The humidity sensing responses for all sensors increased as the RH value increased, indicating that water vapor has a strong influence on conductivity.

**Figure 5:**
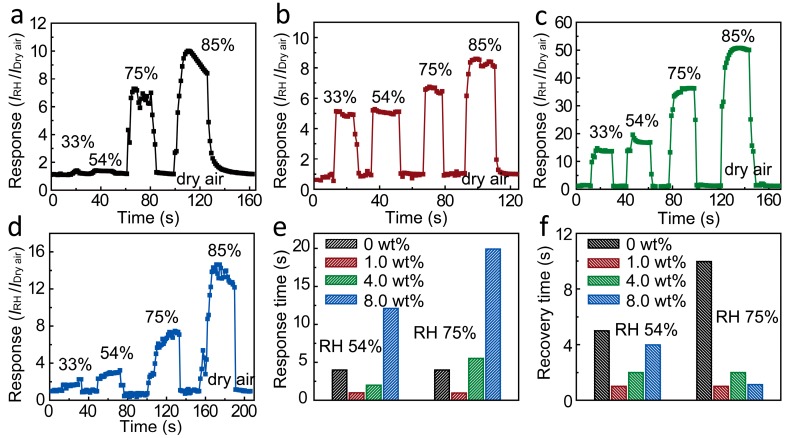
The response and recovery properties at DC 5 V with an Li doping level of (**a**) 0, (**b**) 1.0, (**c**) 4.0, and (**d**) 8.0 wt %, respectively, at different relative humidity values; The comparison of (**e**) the response time and (**f**) the recovery time of different Li^+^-doped SnO_2_ PNFs at relative humidity levels of 54% and 75%, respectively.

The dynamic tests were also characterized to show the water vapor influence on the conductivity of SnO_2_ NWs, as shown in Figure 5a–d. It was found that a conductivity change resulting from the fluctuation of RH is always reversible. When the sensor was exposed to the moist air in reference to dry air, the current promptly increased through the SnO_2_ and then gradually reached a relatively stable value. When the sensor was switched to dry air again, the current abruptly decreased and rapidly reached a relatively stable value. The responses of all sensor materials gradually increased as the RH value increased from 33 to 85%. The results coincided with the response results in Figure 3. The response and recovery behavior is the important characteristics for evaluating the performance of humidity sensors. Figure 5e,f illustrate the response time and recovery time (defined as the time required to reach 90% of the final equilibrium value) comparison under 54% and 75% RH values for different Li^+^-doped SnO_2_ PNFs, respectively. The response time of the SnO_2_ PNFs is ~4 s under both 54% and 75% RH. As Li was doped to SnO_2_, the 1.0 wt % Li^+^-doped SnO_2_ PNFs showed a response time of as low as 1 s under both 54% and 75% RH, much faster than that of 4 s on the pristine SnO_2_ PNFs. The 4.0 wt % Li^+^-doped SnO_2_ PNFs also showed the low response time of ~2 s under a 54% RH value. The recovery time is also an important characteristic for the ultrafast humidity sensor. The recovery time of SnO_2_ PNFs is ~5 s under 54% RH and 10 s under 75% RH. The fast recovery time of 1 s was found on the 1.0 wt % Li^+^-doped SnO_2_ PNFs under both 54% and 75% RH. The 4.0 wt % Li^+^-doped SnO_2_ PNFs also show the fast recovery time of 2 s under both 54% and 75% RH. The Li^+^-doped SnO_2_ PNFs show a recovery time lower than that of the pristine SnO_2_ PNFs. The ultrafast response/recovery speed (within 1 s) of 1.0 wt % Li^+^-doped SnO_2_ nanospheres enables its novel application as a human breathing sensor because human breathing intervals are within 5 s.

The enhanced response, the ultrafast response, and the recovery speed of 1 s could be attributed to the co-contribution of the doping of Li^+^ ions and the 1D characteristic structure of PNFs. The alkaline Li^+^ bonding interface altered the surface correlation with water molecules, which subsequently vary the response and recovery behavior of Li^+^-doped SnO_2_. There is a suitable doping content. Furthermore, the characteristic structure of 1D PNFs has a large surface-to-volume ratio, which makes the absorption of water molecules on the surface of the sensors easy. The 1D nanoporous structure of the fibers can facilitate the aggregation of water molecules and fast mass transfer of the water molecules to and from the interaction region and can improve the rate for charge carriers in PNFs to transverse the barriers induced by molecular recognition along the fibers.

## 3. Materials and Methods

Chemicals were purchased from Aladdin Industrial (Shanghai, China) and used as received without any further purification. Exactly 5.0 wt % of tin (II) chloride dihydrate was dissolved in 40 wt % of N’,N’-dimethylformamide (DMF) and 45 wt % of ethanol under vigorous stirring for 10 min. Subsequently, 10 wt % of poly(vinyl pyrrolidone) (PVP, Mw = 1,300,000, Sigma-Aldrich, Shanghai, China) and a suitable amount of lithium chloride hydrate was added into the above solution under vigorous stirring for 30 min. The mixture was loaded into a glass syringe with a needle of 1 mm in diameter at the tip and was electrified using a high-voltage direct current (DC) supply. Fifteen kilovolts were provided between the tip of the spinning nozzle and the collector at a distance of 20 cm. Finally, the nanofiber mats were peeled off from the collector and placed in a crucible. Calcination (600 °C in air for 5 h) was performed to remove PVP, and get crystallized SnO_2_. The pristine SnO_2_ PNFs were also obtained with a free addition of lithium chloride hydrate by the same procedure.

Most of humidity sensors are investigated in alternating current (AC) conditions by evaluating the impedance change, which can avoid the polarization effects of absorbed water. However, the corresponding signal processing circuits are complicated. In our work, the direct current humidity sensors were fabricated as follows. The pristine and Li^+^-doped SnO_2_ PNFs were separately mixed with deionized water (5:100 by weight) to form a paste, and the paste was then dipcoated onto an 1 cm × 1 cm quartz substrate with a pair of Al electrodes (a length of 2 mm and a gap width of 100 μm). The conductivity of the PNFs was measured using a programmable DC voltage/current generator (Keithley 4200, Tektronix, Inc., Beaverton, OR, USA). The different relative humidity levels were controlled by salt solutions. Two chambers were used in our experiment, and the quick response sensors were measured by switching sensors between the two chambers [17,28,33,34]. Each chamber was stabilized at 25 °C for 12 h to ensure that the air in the chamber reached the equilibrate state, with a standard humidity sensor to monitor the RH in the chambers at the same time. The four different saturated salt solutions were MgCl_2_, Mg(NO_3_)_2_, NaCl, and KCl, were chosen to prepare the atmospheres with the different relative humidity levels. Compared with other salts, MgCl_2_, Mg(NO_3_)_2_, NaCl, and KCl are easy to obtain, stable, and with proper corresponding relative humidity values of 33%, 54%, 75%, and 85% at 25 °C, respectively.

## 4. Conclusions

Pristine SnO_2_ and Li^+^-doped SnO_2_ composite porous nanofibers with various dopant concentrations were fabricated by a simple electrospinning method. The humidity sensing properties of the as-prepared samples were also studied. It was found that the introduction of alkali ion Li^+^ into the one-dimensional SnO_2_ porous nanofiber improves humidity sensing properties. Compared with the pristine SnO_2_ porous nanofibers, the optimized Li^+^-doped SnO_2_ porous nanofibers exhibited an up to 15 times higher response (85% RH) when tested at 5 V and room temperature. Most importantly, the response and recovery times of the Li^+^-doped SnO_2_ porous nanofibers were both below 1 s, which is extremely attractive for ultrafast breathing applications. The high humidity sensitivity, the ultrafast response, and the recovery properties could be attributed to the co-contributions of the doping of Li^+^ ions and the 1D characteristic structure of SnO_2_ porous nanofibers. This work can highly improve the development of Li^+^-doped SnO_2_ porous nanofiber-based direct-current humidity sensors and provide an effective way of developing ultrafast humidity sensors.
